# Time-varying gradient metasurface with applications in all-optical beam steering

**DOI:** 10.1515/nanoph-2022-0756

**Published:** 2023-03-22

**Authors:** Mohammad Karimi, M. Zahirul Alam, Jeremy Upham, Orad Reshef, Robert W. Boyd

**Affiliations:** School of Electrical Engineering and Computer Science, University of Ottawa, Ottawa, ON, K1N 6N5, Canada; Department of Physics, University of Ottawa, Ottawa, ON, K1N 6N5, Canada; The Institute of Optics, University of Rochester, Rochester, NY, 14627, USA

**Keywords:** epsilon-near-zero (ENZ), metasurface, nonlinear optics

## Abstract

Integrating the large, subpicosecond nonlinear optical response of epsilon-near-zero (ENZ) materials with the broad design freedoms of plasmonic metasurfaces shows potential for creating rapidly modulated optical devices with possible applications in telecommunications, sensing, and reactive beam steering. In this work, we experimentally investigate a metasurface consisting of a plasmonic gradient array on a thin layer of indium tin oxide (ITO), characterize how incident probe pulses diffract from a system as it is being dynamically modulated by a pump pulse at wavelengths near the ENZ region. Angular shifts in the diffraction orders are observed and can be principally attributed to the adiabatic wavelength conversion of the probe as it witnesses the temporal change of index induced by the pump. Of note, the asymmetric gradient metasurface, considered to be a blazed diffraction grating, shows significantly different dynamic responses for different diffraction orders. The free-space wavelength shift to +1 and −1 diffraction orders is 6 and 12 nm, resulting in steering angle changes of 0.65 and 1.5°, respectively.

## 1 Introduction

Metasurfaces are 2D arrays of subwavelength scattering objects that can be used to control the vectorial properties of electromagnetic radiation [1]. These structures have been used for many different applications, such as in making flat lenses [2], holograms [3], high-quality factor resonators [4], highly nonlinear platforms [5], and vortex beam generators [6]. An important group of metasurfaces is gradient metasurfaces, where the electromagnetic response of the surface is position dependent.

One application of such structures is to control the wave vector direction of the reflected or transmitted wave from the surface, known as anomalous reflection and refraction, respectively [7]. The underlying mechanism for this phenomenon is that the light that scatters from a subwavelength object experiences some phase shift that depends on the spectral separation between the incident beam's central wavelength and the object's resonance wavelength. If we prepare an array of differently sized scatterers that, as a result, have different resonance wavelengths, the incoming field experiences a position-dependent phase shift at the surface. This position-dependent phase shift produces an additional transverse *k*-vector, which consequently leads to an altered deflection angle of the scattered field. In the case of a periodic pattern of differently sized antennas, the metasurface will behave very similar to a grating that diffracts the incident light into different diffraction orders (DOs). The angle of diffraction for the first DOs, DO = +1 or −1, is determined by the ratio between the wavelength and the lattice size (Λ) through the equation

(1) $$\sin\left(\theta_{t}^{\pm 1}\right)=\sin\left(\theta_{r}^{\pm 1}\right)=\pm \frac{\lambda_{0}}{\Lambda }.$$

The portion of the incident power that couples into each diffraction order at each wavelength depends on the phase distribution over each unit cell [8].

A challenge that has attracted much attention in recent years is how to make time-varying metasurfaces that dynamically control the vectorial properties of scattered light [9–14]. To this end, the metasurfaces are typically embedded within some host medium with optical properties that can be controlled dynamically. Many different host media have been proposed for this purpose, such as mechanically elastic materials [10], liquid crystals [11], phase-change materials [12], semiconductors [13], and transparent conduction oxides (TCOs) [14]. Among these media, elastic materials, liquid crystals, and phase-change materials provide comparatively slower mechanisms with response times in the order of milliseconds or longer [10–12]. One can have much faster control over the optical characteristics of the system by applying a voltage through a gate to the host media made of semiconductors or TCOs and manipulating the electron distribution within these materials [13, 14]. This method, although providing the possibility of microsecond control over the system, requires connecting subwavelength scattering objects to external voltage sources, which may lead to design limitations and fabrication complications. Also, in metasurfaces that are dynamically controlled by an external voltage, the nanoantennas themselves are usually playing the role of the gate, which limits the application of this method to metallic metasurfaces.

To achieve high-speed control over the optical characteristics of a system and to also create an environment to engineer the phase profile of the surface, one may find it useful to design an all-optically controlled scheme to manipulate the optical properties of the system. This would require that a material with high nonlinear optical responses host the metasurface. Incorporating such material would enable changes to the optical properties of the system by applying a pump field of sufficient intensity to trigger the nonlinear response of the host medium.

Indium tin oxide is a TCO with a permittivity that follows the Drude model in the infrared (IR) region [15]. The real part of ITO permittivity vanishes at a wavelength in the IR range called the ENZ point [15]. When illuminating a thin layer of ITO with a sufficiently intense laser pulse, the electrons undergo an intraband energy level transition in a subpicosecond time scale, leading to a large and fast refractive index modification of ITO. Thin films (310 nm) of ITO are known to possess one of the largest ultrafast nonlinear refractive indices among solid materials [16]. This nonlinear response can be enhanced further when the ENZ mode within the ITO layer couples to the electric dipole resonance of an array of plasmonic nanoantennas [5]. This enhancement will occur when the resonance wavelength of the antennas is close to the ENZ point of the ITO layer such that the ENZ mode strongly couples to the electric dipole mode of the metasurface [17].

An exciting application of the large and fast nonlinear response in ITO, or the coupled system of ITO and a plasmonic metasurface, is adiabatic wavelength conversion (AWC) [18]. In this process, an intense pulsed laser pump beam induces a nonlinear response in the system, generating a refractive index change that rises considerably and then falls back to the initial value within a subpicosecond time duration. A low-intensity laser probe beam may interact with the time-varying response caused by the pump and depending on their relative timing, the probe may observe a rising $\left(\frac{\Delta n}{\Delta t}>0\right)$ or falling $\left(\frac{\Delta n}{\Delta t}<0\right)$ refractive index response, leading to a red-shift or blue-shift of the probe’s wavelength, respectively [18]. Since the index rise time is shorter, the magnitude of the red-shift is larger.

Our goal has been to design and fabricate a gradient metasurface with gold nanoantennas over a thin layer of ITO to optically manipulate the light diffracted by the metasurface. The device is tested in a pump-probe setup providing a system for the all-optical control of the spectral and vectorial distribution of the probe beam DOs. For an appropriate time delay between the pump and the probe, the free-space wavelength of the diffracted beam would be shifted and so the deflection angle for DO = ±1 will change based on Eq. (1) and its following discussion. This system can be considered to be an all-optical beam steering device.

## 2 Metasurface design

We designed the metasurface as a repeating group of nanoantennas of different lengths to provide an overall response akin to a blazed diffraction grating while ensuring that each antenna showed good coupling to the ITO substrate. This requirement considers both the dimensions of each antenna in a unit cell and the properties of the ITO substrate.

The selected ITO thickness (65 nm) has an ENZ mode that can couple to electric dipole resonance modes of the nanoantennas and provides the best trade-off between the diffraction efficiency and the strength of the nonlinear response. The ENZ mode is a naturally dark mode that exists in very thin layers of ENZ material sandwiched between two dielectrics [19]. The ENZ point for our ITO layer is 1360 nm (Figure S1 of the supplementary information). The value of the imaginary part of the permittivity of ITO at the zero-permittivity wavelength of 1360 nm is 0.35. Thus, the 65-nm-thick ITO films considered in this work have a non-negligible absorption loss of around 20% in our wavelength range of interest. We require that the electric dipole moment of each antenna resonate near the zero-permittivity wavelength while also differing from the resonance wavelength of the adjacent nanoantennas. Therefore, all the dipole moments couple to the ENZ mode of the ITO substrate efficiently while contributing to creating the gradient metasurface. The wavelength of the peak of the ENZ mode may be found by solving the wave equation in the thin ENZ layer sandwiched between a glass substrate and air superstrate [19]. That wavelength can be slightly different from the ENZ wavelength, but this does not affect our design procedure.

Each unit cell consists of four antennas with identical widths but different lengths. To select the lengths of the antennas, we simulated uniform metasurfaces made of identical antennas using the finite difference time domain (FDTD) method and after sweeping over the length of the antennas, recorded the phase of the reflected and transmitted field for each length. Since the antennas exhibit a broader range of phases in reflection than in transmission, we designed our metasurface to apply the largest linear phase ramp over a unit cell on the reflected field. The distance between adjacent antennas (344 nm) is sufficient to neglect the coupling between neighboring antennas, which is essential in our design as we require the resonance behavior of individual antennas to be the same in the final gradient metasurface as it would be in a uniform metasurface. The number of antennas in each unit cell makes the unit cell size slightly larger than the wavelength of interest, thus maximizing the steering angle of the 1st DOs in response to wavelength conversion (see Eq. (1)), as explained in the following.

**Figure 1:**
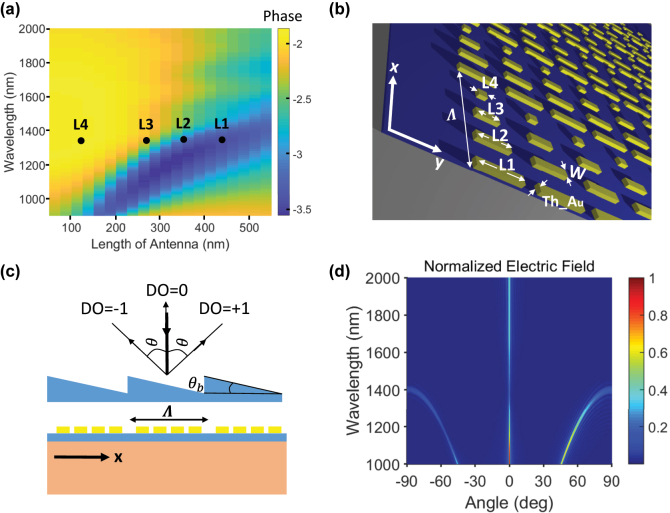
The design procedure of the plasmonic metasurface. (a) The phase of the reflected field from a metasurface of homogeneous gold antennas of a constant thickness (50 nm) and width (90 nm). The four selected lengths (L1 to L4) are indicated with black dots. (b) 3D schematic of the final design of the device; the dimensions in the figure are L1 = 420 nm, L2 = 330 nm, L3 = 270 nm, and L4 = 100 nm. The period along the gradient is Λ = 1375 nm, and the separation between rows is *P* = 600 nm. (c) Schematic demonstrating the similarity of a periodic gradient metasurface and a blazed grating. (d) FDTD simulation of the angular distribution of the +1, 0, and −1 DOs of reflection at different wavelengths.

Figure 1(a) shows the phase of the reflected field from a metasurface of homogeneous antennas of constant thickness and width but different lengths across different wavelengths simulated by Ansys Lumerical's FDTD software. The maximum phase ramp for a wavelength around the ENZ point is at an operating wavelength of 1300 nm. The black dots indicate the lengths of the nanoantennas selected for the final gradient metasurface such that they provide the maximum phase ramp on the reflected field over each unit cell. Figure 1(b) represents the schematics of the designed structure. Four antennas of thickness = 50 nm, width = 100 nm, and lengths = 440, 330, 270, and 100 nm were selected to make a unit cell of size 1375 nm by 600 nm for the final design. With the selected lattice size, ±1 DOs deflect to an angle of *θ* = 71° (as in Figure 1(c)) under normal incidence of the incident beam, as can be derived using Eq. (1). Figure 1(d) shows the angular dispersion of the DOs of reflection for the gradient metasurface. The metasurface primarily diffracts light toward the +1 DO as it is a blazed grating. However, the metasurface also diffracts a non-negligible amount of power in the −1 DO direction primarily due to phase and digitization errors introduced by the antennas.

## 3 Results and discussion

**Figure 2:**
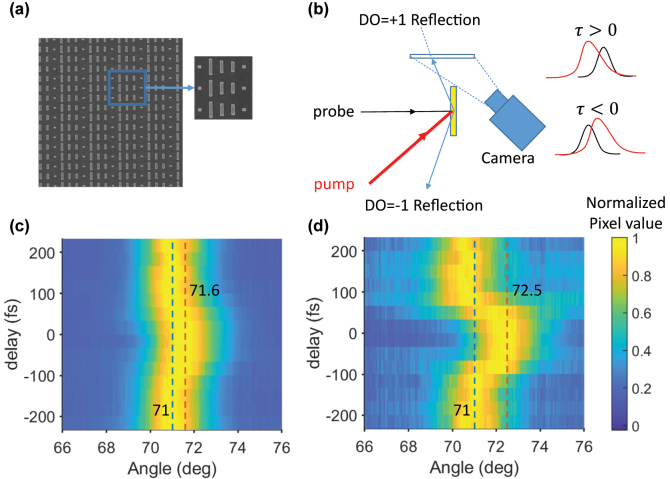
Fabrication and characterization of the sample. (a) The SEM image of the fabricated device. (b) A simple schematic of the pump-probe set-up with a camera to image the diffracted beams at different time delays between the pump and the probe. (c) and (d) The angular distribution of DO = +1 and DO = −1 of the probe, respectively, at different time delays between the pump and the probe. The power of each row (each delay) is normalized to the brightest pixel in that row.

Device fabrication used a standard lift-off process on a sample of 65-nm-thick ITO on glass. Two layers of PMMA with different molecular weights, 495PMMA and 950PMMA, were spun over the sample as the bottom and the top resist layers, respectively. The layout of the metasurface was then written into the resist using electron beam lithography followed by a development of the sample in MIBK/IPA 1:3. A 27-nm-thick gold layer was then evaporated onto the sample using a thermal source. Finally, the excess gold over the resist lifted off the sample after laying inside 70 °C acetone overnight. Figure 2(a) shows an SEM image of the final fabricated device. We used a degenerate pump-probe setup to test the sample. The pump and probe pulses are 120 fs in duration with a repetition rate of 1 KHz, generated together by a tunable optical parametric amplifier driven by a Ti:sapphire source and then split and routed to the metasurface, as shown (Figure 2(b)). The probe illuminates the sample from the ITO side at normal incidence and a constant, low intensity of 100 MW/cm^2^. The reflectance of the pump from the metasurface increases with increasing angles of incidence (Figure S3). Thus, to avoid large reflectance of the pump, we choose a small (5°) angle of incidence, which appeard to produce the best response.

We could distinguish DO = ±1 of the probe in reflection and transmission. While our measurements focus on the reflected DOs, we observed similar responses for the transmitted DOs. We placed an observing screen in the path of each of the DOs and then imaged the screen onto an IR camera. We repeated this procedure for DO = ±1 as a function of the time delay between the pump and the probe.

Figure 2(c) and (d) show the angular distribution of the power of DO = ±1 for different time delays between the pump and the probe. A negative time delay means that the probe precedes the pump. The central wavelength of the incident light is 1300 nm, and the intensity of the pump is 14 GW/cm^2^. The results for different wavelengths and intensities will be presented and discussed later. We see that the DO power is distributed around a central angle of 71°. When the pump and the probe overlap in time, the diffraction angles of the DOs change. The maximum steering occurs at a negative time delay of −33 fs.

Of particular interest is the surprising result that the magnitude of steering is 0.6° for DO = +1 and 1.5° for DO = −1, suggesting that these two diffraction orders respond significantly differently to the temporal change of the refractive index. This asymmetric response was not anticipated but is consistent and repeatable. It could not be readily explained by asymmetries of the characterization stage; while the pump beam illuminates the sample at an angle, switching between the DO = +1 and −1 sides by loading the sample upside down or interchanging the pump and the probe beams produced the same asymmetric nonlinear behavior of the DOs. This asymmetry of steering response was also consistent across separately fabricated metasurfaces, as long as they followed this same design. Similarly, the difference in response holds for different input powers and wavelengths. Therefore, for this metasurface design, the DO = −1 diffraction order shows a consistent steering response that is roughly twice as large as its DO = +1 counterpart.

Having dismissed these other possible causes for this asymmetric response in beam steering, we are left to conclude that it is caused by the asymmetry of the metasurface itself, which is to say, the blazing angle imposed by the asymmetry of the nanoantenna placement within each unit cell of the metasurface. One possible explanation is that because the blazing frustrates the coupling of the probe light to the −1 diffraction mode through built-up interference of the different nanoantennas, light that does ultimately couple to this less preferred mode has spent more time in the metasurface compared to light diffracting in the DO = +1 mode. If so, it would consequently have witnessed more of the temporal change of refractive index induced by the pump. Should this be the case, we would predict that the light diffracted to the DO = −1 mode would have not only a larger change in angle but also a larger change in the spectrum as this mode has undergoes a greater degree of AWC by the pump. Also, to compare the interaction time of different DOs with the metasurface and confirm the longer interaction time of DO = −1, we extracted the phase accumulated by each diffraction order over the interaction with the metasurface from the Comsol Multiphysics simulation of the device in the linear regime. Figure S5 in the supplementary shows this phase for different reflected DOs and demonstrates that the phase accumulation over the metasurface for DO = −1 is almost two times larger than that of DO = +1 in our wavelength range of interest. This phase accumulation suggests that DO=−1 has a longer interaction time with the metasurface. It is also noteworthy that a similar asymmetric nonlinear response of different scattering modes in ENZ media has also been reported by Bruno et al. [20].

**Figure 3:**
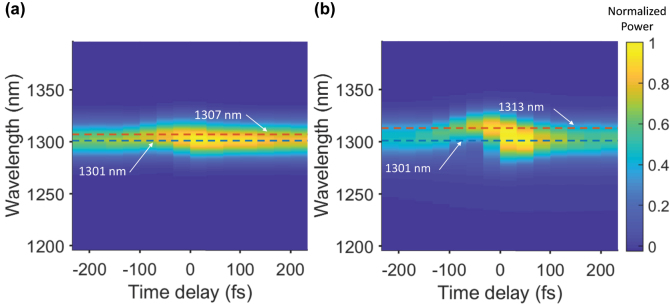
The spectral distribution of the (a) DO = +1 and (b) DO = −1 for different time delays between the pump and the probe. Here power is normalized to the brightest value in the whole graph.

In order to confirm that AWC is the principal mechanism for the steering of the beam, we measured the spectrum of different portions of the beams that diffracted to different angles with respect to the time delay between the pump and the probe. Figure 3 demonstrates the wavelength distribution of DO = ±1 for the incident-field central wavelength of 1300 nm at a constant 14 GW/cm^2^ pump intensity and for different time delays between the pump and the probe. In order to create this graph, we measured the spectra of DO = ±1 separately for different diffraction angles and then integrated the data over these angles to create a 1D wavelength-dependent data for each time delay. The largest wavelength shift happened for the delay time of −33 fs and was 6 nm for DO = +1 and 12 nm for DO = −1. These magnitudes of wavelength conversion reflect the proportional changes to the steering angles of 0.8 and 1.6° using Eq. (1) for DO = +1 and DO = −1, respectively, and are in good agreement with the camera data discussed above.

**Figure 4:**
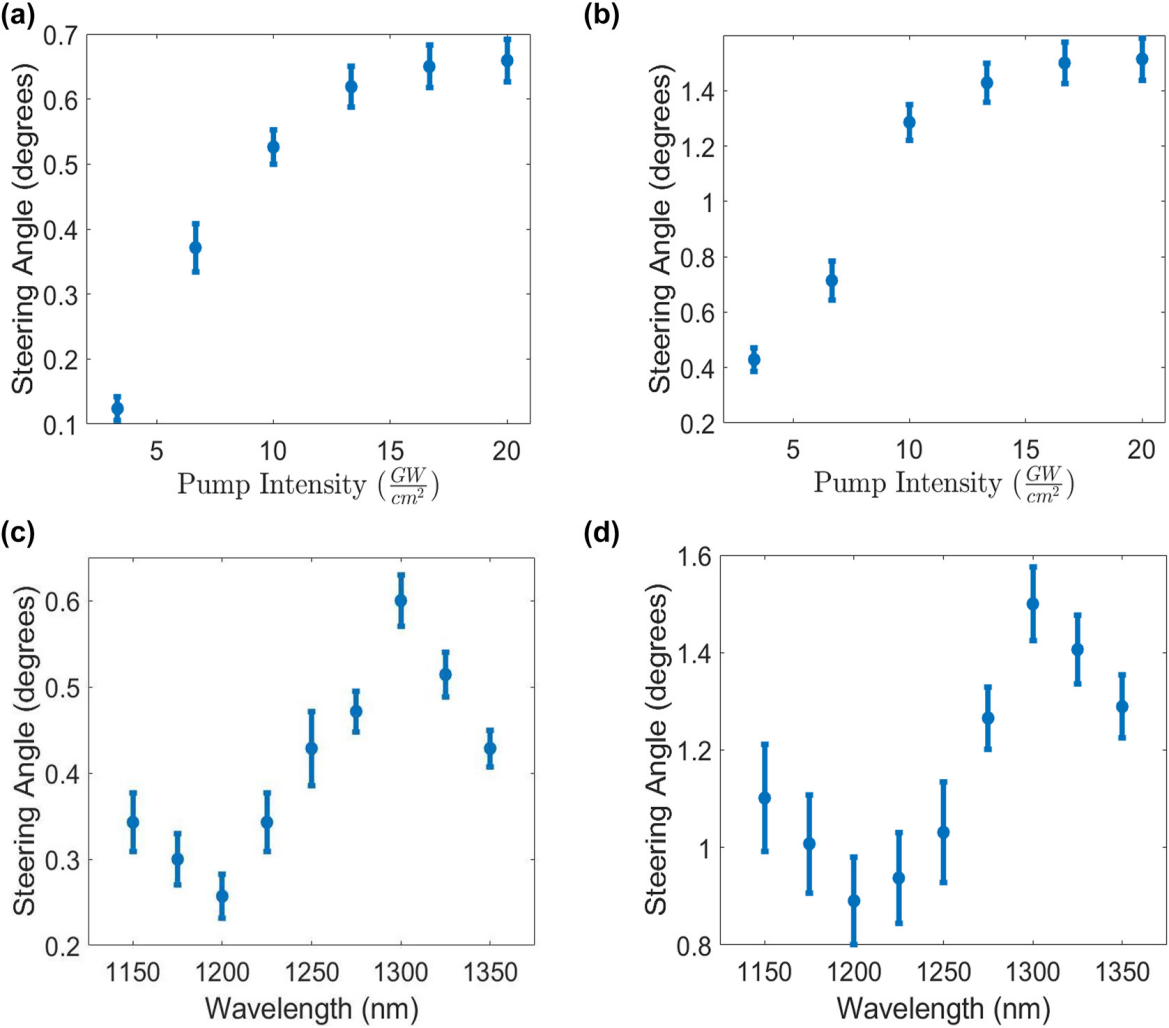
The maximum steering angle at different incident wavelengths and powers. (a) and (b) Show the maximum steering angle of DO = +1 and DO = −1, respectively, at different pump intensities for an incident central wavelength of 1300 nm. (c) and (d) Show the maximum steering angle of DO = +1 and DO = −1, respectively, for different wavelengths of the incident beams while the intensity of the pump is kept constant at 14 GW/cm^2^.

We also investigated the effect of the pump power on the steering angle of the DOs of the probe. Figure 4(a) and (b) show the maximum steering angle for the incident field central wavelength of 1300 nm at different pump intensities. The steering angle increases with intensity for up to 14 GW/cm^2^ and then starts to saturate. This saturation can be attributed to the limited capacity of the upper levels in the conduction band of ITO that limits the intraband transitions, which are the primary source of nonlinearity in ITO [16]. The damage threshold of our sample is approximately 20 GW/cm^2^ and is dictated by that of the gold nanoantennas. This value is consistent with previous reports [21]. Note that the asymmetry between DO = +1 and DO = −1 is observable in all powers of the pump.

It is crucial to study the response of the device at different wavelengths. This can be investigated in two different regimes: linear and nonlinear. The linear response of the antennas has wavelength dependence, so the phase ramp over each unit cell will be modified, as will the distribution of power that couples into the DOs. Similarly, the nonlinear response is also wavelength dependent, as we expect a larger nonlinear response when we are near the resonance wavelength of at least one of the antennas. The closer and stronger the resonance, the larger the confinement of light in the nonlinear substrate. As a result, the spectral response of the device is mainly influenced by the resonance properties of the nanoantennas. Figure 4(c) and (d) show the maximum steering angles for different central wavelengths of the incident field while the pump power is kept constant. The maximum steering angle for both DOs happens at a probe central wavelength of 1300 nm. While the maximum nonlinearity for a bare sample of ITO occurs around the ENZ wavelength, when we place nanoplasmonic antennas on top of the ITO, the maximum nonlinearity wavelength blue-shifts [5]. One observes that the asymmetric response of DO = +1 and DO = −1 holds for all wavelengths measured.

Going forward from this proof-of-principle experiment, there are a few design approaches to increase the observed angular shifts. First, the observed angular shift's magnitude is proportional to the wavelength change's magnitude, so increasing the achievable AWC can be considered. Larger AWC has been reported [18] in an ENZ material and could be presumably improved by increasing the interaction time between the probe and metasurface during the latter’s nonlinear response to the pump. Second, this metasurface was designed for a large phase ramp across the unit cell to maximize the diffraction efficiency in the linear regime rather than to produce optimal coupling to the ENZ material. As a consequence, some of the antennas resonate away from the ENZ wavelength and will have reduced nonlinear enhancement. Designing for a different trade-off between the power coupling efficiency and the strength of the nonlinear response could increase the AWC and thus the angular swing. Third, if the metasurface incorporated a back-reflecting plane below the ENZ material, it would likely improve both the nonlinear phase shift and the power coupling efficiency, thus increasing the range of angular shift.

In conclusion, we propose an all-optical scheme to make a dynamic gradient metasurface using the large and subpicosecond nonlinear response of ITO around its ENZ wavelength. We designed a periodic gradient plasmonic metasurface consisting of an array of gold nanoantennas over a 65-nm ITO layer. The incident light diffracted as expected to different DOs in both reflection and transmission with the diffraction angle dependent on the free-space wavelength and the periodicity of the array. We induced the large, fast nonlinear response of the ITO-based metasurface, leading to adiabatic wavelength conversion (AWC) of the diffracting beam, and the converted wavelengths diffracted to new angles. In other words, this scheme uses an optically pumped AWC effect to steer the DOs of a light beam to different angles. This effect was studied in detail for the reflected DOs, but similar responses are achievable in transmission.

The maximum amount of wavelength conversion and steering angle was different for the cases of DO = +1 and DO = −1. A question that remains is whether the asymmetry of the metasurface which produce asymmetry in the frequency shift and thus also the magnitude of beam steering is a designable parameter. For instance, how would the relative magnitude of the shift of these two diffraction orders change with blazing angle? Would the relative magnitude of shift be proportional to the relative diffraction efficiency of these two diffraction orders? Finally, how would this asymmetry DO behave for higher order (2nd, 3rd) diffraction modes? These questions all require the design, fabrication, and characterization of new asymmetric metasurfaces and are, therefore, beyond the scope of this report but may be an intriguing avenue for further study.

Finally, the scheme for dynamic control of light scattering from a metasurface is entirely compatible with metasurfaces of different antenna shapes or different materials, including dielectrics. Hence, opportunities exist to expand functionality via holography or explore different wavelength ranges.

## Supplementary Material

Supplementary Material Details
