# Supplementary material for: Time-varying gradient metasurface with applications in all-optical beam steering
Source: Nanophotonics. 2023 Mar 22;12(9):1733–40. doi: 10.1515/nanoph-2022-0756 (PMC11501296; doi:10.1515/nanoph-2022-0756)
Supplement: Supplementary file 1 — Supplementary Material Details [file j_nanoph-2022-0756_suppl_001.pdf]

Research Article

Mohammad Karimi*, M. Zahirul Alam, Jeremy Upham, Orad Reshef, and Robert W. Boyd

Supplementary Material: Time-varying gradient metasurface with applications in all-optical beam steering

https://doi.org/10.1515/sample-YYYY-XXXX
Received Month DD, YYYY; revised Month DD, YYYY; accepted Month DD, YYYY

Abstract: This document contains supplementary information for the paper entitled "Time-varying gradient metasurface with applications in all-optical beam steering". In Section 1 we provide the ellipsometry data for the wavelength-dependent permittivity of the ITO sample we used around the ENZ point. Section 2 shows the angular distribution of the reflected diffraction orders from the surface alongside the phase that is accumulated by the reflected diffraction orders. Finally, Section 3 shows the schematic of the set-ups that used for testing the device.

Keywords: Nonlinear Optics, Epsilon-Near-Zero (ENZ), Metasurface

1 The permittivity of the ITO sample

The permittivity of ITO in the infrared region follows the Drude model as in figure S1. The relation between the permittivity and the frequency in the Drude model can be represented as

$$\epsilon(\omega) = \epsilon_\infty + \frac{\omega_p^2}{\omega(\omega + i\gamma\omega_p)} \tag{1}$$

where $\epsilon_\infty = 3.69$, $\omega_p = 2.67e^{15} rad/s$ and $\gamma = 0.045\omega_p$ for our ITO sample.

2 Electric field enhancement

In order to investigate the enhancement of the field within ITO, induced by the nanoantennas, we plot the electric field enhancement at the central wavelength of 1300 nm in figure S2. The data was taken from the simulation of the structure in Lumerical FDTD. We observe that each antenna is resonating differently at this specific wavelength and so we have different field enhancement around each antenna.

***Corresponding author: Mohammad Karimi,** School of Electrical Engineering and Computer Science, University of Ottawa, Ottawa, ON, K1N 6N5, Canada, e-mail: karimimohammad1991@gmail.com
M. Zahirul Alam, Jeremy Upham, Orad Reshef, Robert W. Boyd, Department of Physics, University of Ottawa, Ottawa, ON, K1N 6N5, Canada
Robert W. Boyd, The Institute of Optics, University of Rochester, Rochester, NY, 14627, USA

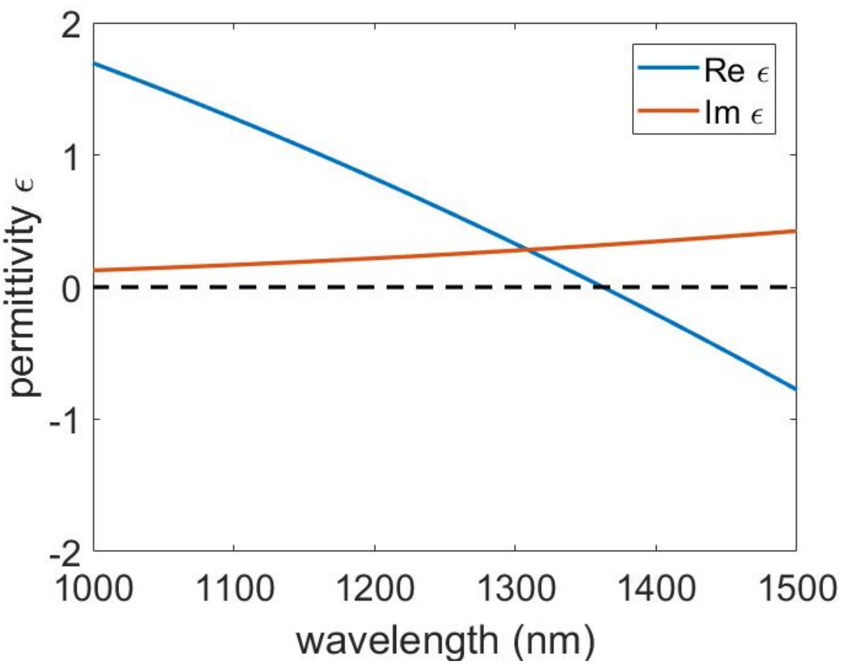

Fig. S1: The permittivity of the ITO sample with a thickness of 65 nm used in our experiment extracted from ellipsometry and fit using the Drude model.

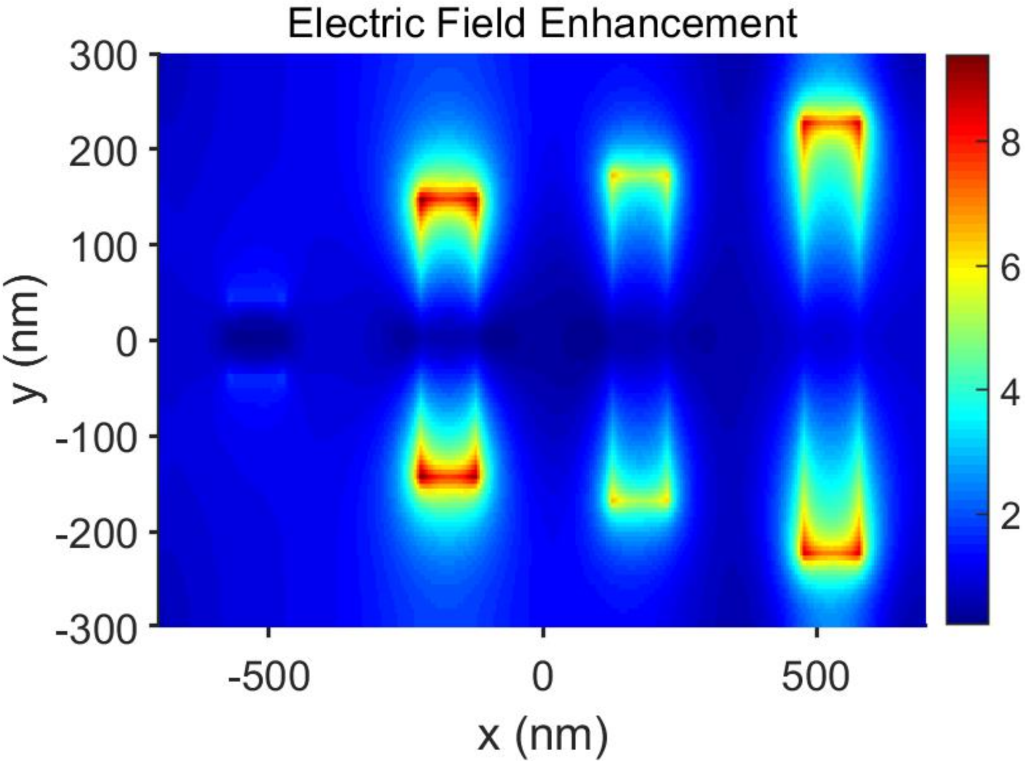

Fig. S2: The enhancement of the electric field in ITO adjacent to the nanoantennas in a single cell at wavelength = 1300 nm. The simulation was done in Lumerical FDTD.

3 The angular distribution of the diffraction orders

3.1 effect of ITO on the angular distribution

Due to the non-negligible imaginary part of the ITO permittivity, the presence of ITO will introduce more loss to the system. It also affects the effective refractive index of the system that leads to the manipulation of the power distribution between different DOs. Figure S3 compares the angular distribution of the DOs in the absence and the presence of 65-nm layer of ITO.

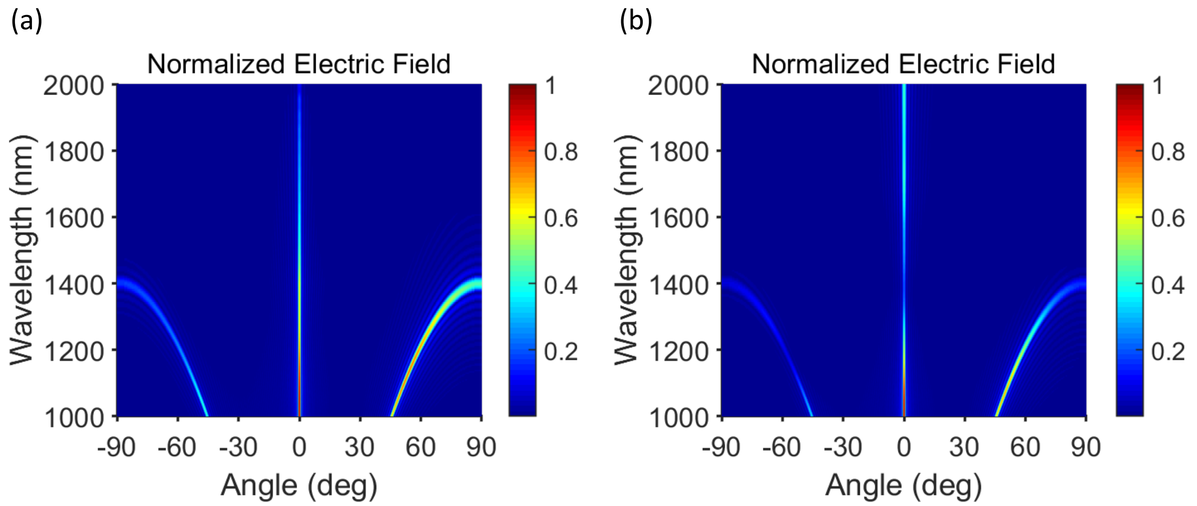

Fig. S3: The distribution of the norm of electric field to different angles and at different wavelengths in reflection. (a) is in the absence of the ITO substrate and (b) is in the presence of the ITO substrate

3.2 Total reflectance at different angles of incidence

In order to give an insight on how the beams are interacting with the material at larger angles we simulated the structure under different angles of incidence. Figure S4 shows the total reflectance of the beam as a function of incident angle inside the glass. We see that the reflectance goes up at larger angles of incidence which affects the portion of light that interacts with the system.

3.3 Spectral response of diffraction orders

To investigate the portion of the incident power that couples into each diffraction order (DO), we performed some linear simulations and experimental characterizations. Figure S5 shows the coupling efficiency of different diffraction orders in (a) reflection and (b) transmission for our sample. The solid lines are the simulation results using COMSOL Multiphysics and the dots with error bars are the experimental results. It is also important to study the phase of the reflected DOs accumulated over the metasurface to get an

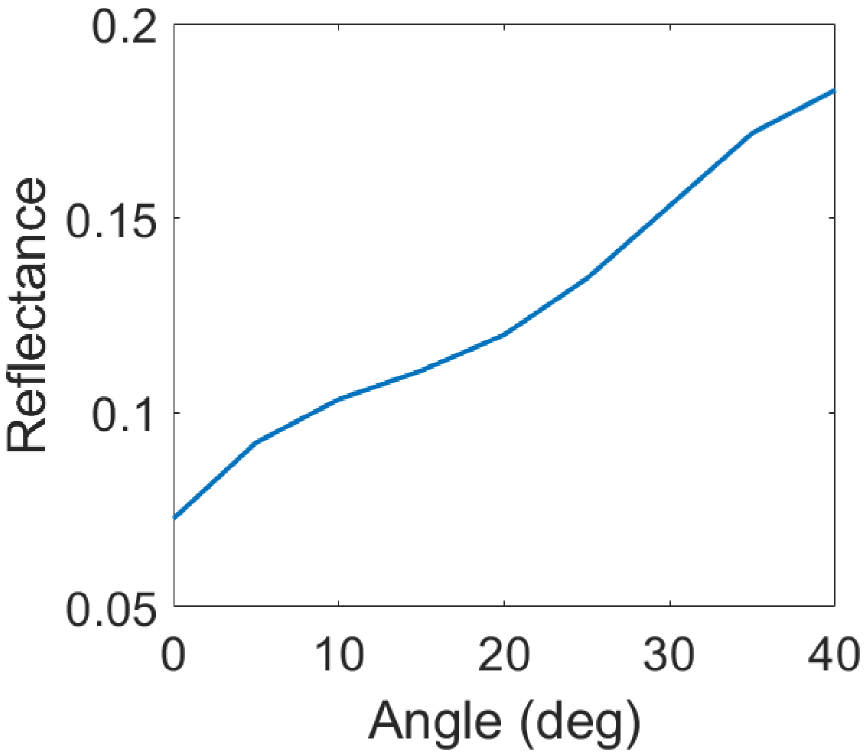

Fig. S4: The total reflectance of the beam from the sample at different angles of incidence within the glass

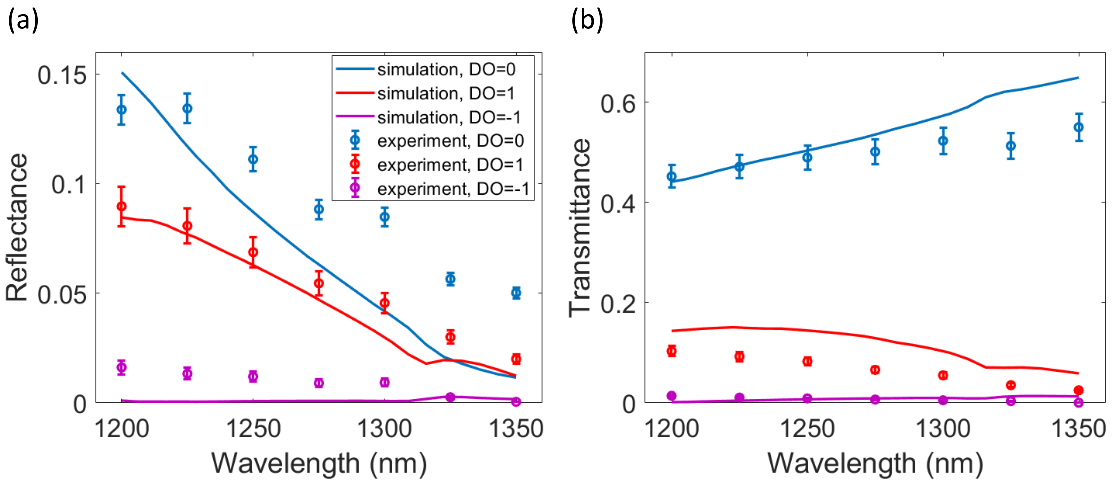

Fig. S5: The linear characterization of the sample in reflection (a) and transmission (b), and in simulation (solid lines) and experiment (error bar dots) for diffraction orders DO=0,±1. The vertical axis in both plots shows the portion of the incident light that has coupled to the diffraction orders.

estimation of the interaction times of each DO with the medium. Figure S6 shows the accumulated phase for the reflected DOs. The phase plotted in this figure is extracted from the simulation of the device in Comsol Multiphysics after the subtraction of the accumulated phase from the port to the surface and vice versa. It suggests that the phase acquired by DO=-1 after interacting with the medium is almost twice larger than that of DO=+1 which can be translated into a longer interaction time of the former DO with the time-varying medium. These results can, at least qualitatively, approve our explanation for the asymmetric nonlinear response of the device demonstrated in figures 2, 3, and 4 of the paper. Note that this phase accumulation result is performed in a linear regime and is not expected to explain all the results that we observed in the nonlinear regime quantitatively as it has to be superimposed by the nonlinear response of ITO/plasmonic metasurface system at different wavelengths to give us a complete insight on the performance of the device.

Figure S7 (a) shows that how the periodic gradient metasurface can be modeled as a blazed grating. When the designed metasurface is illuminated at normal incidence, two diffraction orders of DO=+1 and DO=-1 are diffracted to two angles symmetric around the normal of the surface. Figure S7 (b) shows how the power is distributed between different angles at different wavelengths for the linear case. Figure S7 (d) shows the same results with the plasma frequency of ITO in the Drude model shifted by 10 %. This amount of shift in plasma frequency has been found to be a good approximation to model the change in the permittivity due to nonlinear responses with our pump intensity [1, 2]. The results in this figure show how the distribution of power between different wavelengths and angles can change due to time-independent nonlinear effects such as the modification of the phase ramp over each unit cell of the metasurface. We see that the time-independent effects are small enough to be ignored in comparison to adiabatic wavelength conversion (AWC).

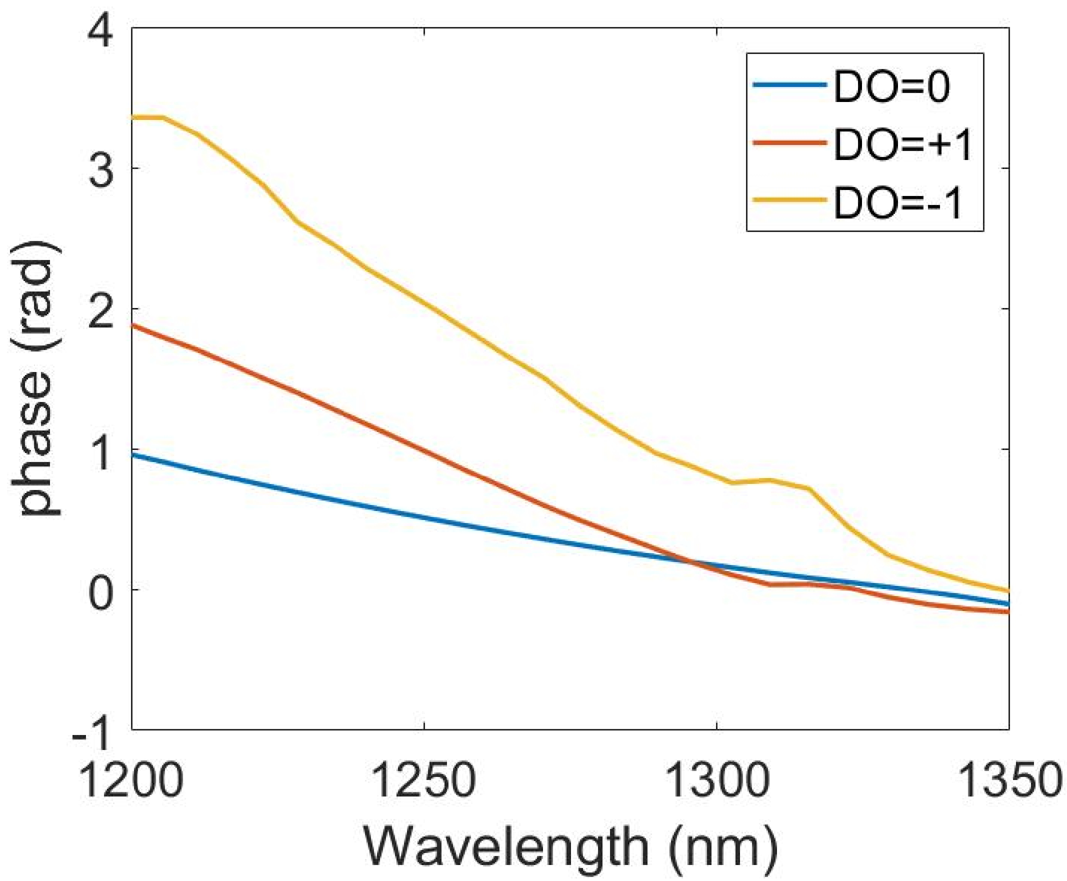

Fig. S6: The phase gained by each reflected diffraction order from the metasurface simulated using Comsol Multiphysics.

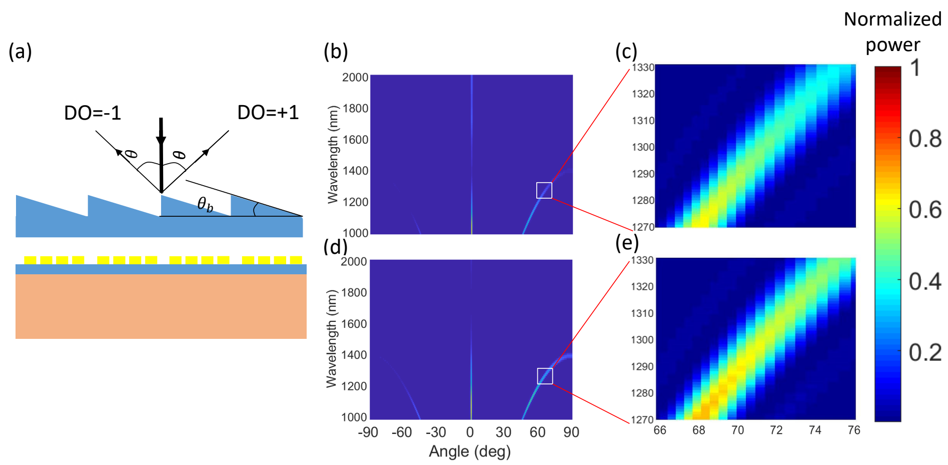

Fig. S7: (a) The schematic of a blazed grating as a model to our gradient metasurface. DO=+1 and DO=-1 are the first diffraction orders. (b) and (c) The angular distribution of DO=+1 for the linear and static nonlinear cases respectively.

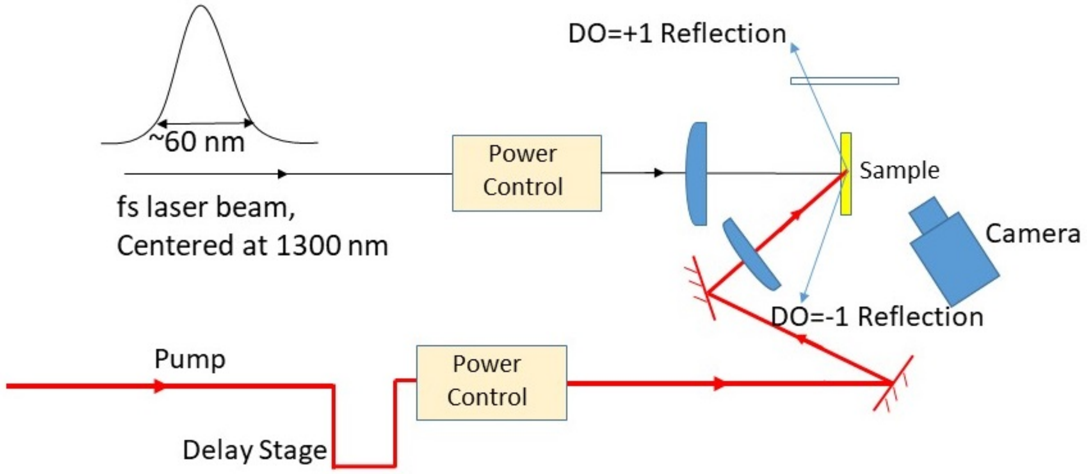

Fig. S8: The schematic of the set-up for measuring the angle distribution of the power for different diffraction orders.

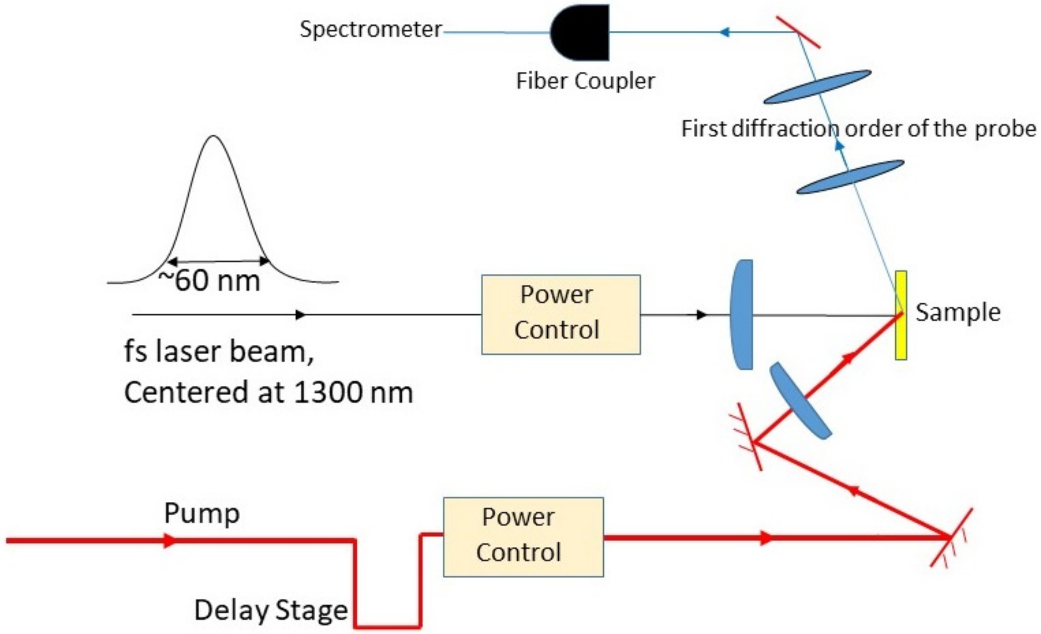

Fig. S9: The schematic of the set-up for measuring the angle-dependent spectrum of different diffraction orders.

4 Measurement set-ups

We used a standard degenerate pump-probe set-up to characterize the sample. The duration of our pulse has been measured to be around 120 fs. The spectral width of our beam is around 60 nm at a central wavelength of 1300 nm. A delay line has been inserted in the pump path to finely control the time delay between the pump and the probe beams. The probe is illuminating the sample at normal incidence and the incident angle of the pump has been set to 5 degrees. Figure S8 shows the schematic of the set-up used

to measure the angular distribution of power using a screen and an infrared camera. Figure S9 shows the scheme of the set-up used for measuring the spectrum of the diffraction orders at different angles.

References

[1] M. Z. Alam, I. De Leon, and R. W. Boyd, “Large optical nonlinearity of indium tin oxide in its epsilon-near-zero region,” *Science*, vol. 352, no. 6287, pp. 795–797, 2016.
[2] M. Z. Alam, S. A. Schulz, J. Upham, I. De Leon, and R. W. Boyd, “Large optical nonlinearity of nanoantennas coupled to an epsilon-near-zero material,” *Nature Photonics*, vol. 12, no. 2, pp. 79–83, 2018.
